# Dementia Literacy among Community-Dwelling Older Adults in Urban China: A Cross-sectional Study

**DOI:** 10.3389/fpubh.2017.00124

**Published:** 2017-06-07

**Authors:** Haifeng Zhang, Samantha M. Loi, Shu’aijun Zhou, Mei Zhao, Xiaozhen Lv, Jing Wang, Xiao Wang, Nicola Lautenschlager, Xin Yu, Huali Wang

**Affiliations:** ^1^Dementia Care and Research Center, Peking University Institute of Mental Health (Sixth Hospital), Beijing, China; ^2^Beijing Key Laboratory for Translational Research on Diagnosis and Treatment of Dementia, Beijing, China; ^3^National Clinical Research Center for Mental Disorders (Peking University Sixth Hospital), Beijing, China; ^4^Academic Unit for Psychiatry of Old Age, Department of Psychiatry, The University of Melbourne, Parkville, VIC, Australia; ^5^Aged Persons Mental Health Program, North Western Mental Health, Melbourne Health, Parkville, VIC, Australia; ^6^Institute of Medical Education, Peking University Health Science Center, Beijing, China

**Keywords:** dementia, literacy, elderly, urban population, China

## Abstract

**Objective:**

Delay in seeking diagnosis of dementia is common in China. Misinformation and poor knowledge about dementia may contribute to it. The study was designed to explore the nationwide dementia literacy among older adults in urban China and to investigate the factors associated with overall dementia literacy.

**Methods:**

In a cross-sectional study, a convenience sample of 3,439 community-dwelling old adults aged 60 and over was recruited from 34 cities in 20 provinces between June 20 and August 20, 2014. All participants were administered the face-to-face mental health literacy questionnaire, which included the prevalence, symptoms, intention, and options for treatment of dementia. Stepwise multivariate regression analysis was used to explore factors associated with overall dementia literacy.

**Results:**

The response rate was 87.4%. The overall dementia literacy was 55.5% (SD = 20.9%) among all respondents. The correct response rate was higher for questions on symptoms (58.7–89.6%), but lower for questions on the prevalence (22.2%) and choosing appropriate professional care personnel (22.2%). Being male [*OR* = 1.256, 95% CI (1.022–1.543)], having lower per capita annual income [OR = 1.314, 95% CI (1.064–1.623)], lower education [OR = 1.462, 95% CI (1.162–1.839)], and suspected depression [OR = 1.248, 95% CI (1.009–1.543)] were negatively associated with overall dementia literacy.

**Conclusion:**

Dementia literacy among community-dwelling older adults in urban China remains very low, in particular about the impact of dementia and appropriate treatment personnel. Community educational programs aiming to close this knowledge gap are encouraged to focus on those in the population at highest risk of low dementia literacy.

## Introduction

Dementia is a progressive, neurodegenerative condition that causes deterioration in memory and other cognitive domains that influence a person’s functioning. The most common type of dementia is Alzheimer’s dementia, which makes up 50–70% of cases. Other common types include vascular dementia, dementia with lewy bodies, and frontotemporal dementia (1). With the global aging of population, the number of people with dementia (PWD) is increasing worldwide. According to the World Alzheimer’s Report 2016, the worldwide prevalence of dementia among old adults aged 60 years and above is 5.2%. This means that about 46.8 million people now live with dementia globally, and this number will almost double every 20 years, to 74.7 million in 2030 and 131.5 million in 2050. Moreover, nearly 25% of them reside in China (2). Thus, China is now facing significant challenges with regard to early recognition, timely diagnosis, and best possible management of dementia.

Despite the current absence of a cure for dementia, it is important for people to have access to early assessment and timely diagnosis. In China, the level of undiagnosed dementia remains high (3). PWD and their families often do not seek help until symptoms are moderate or severe. Zhao et al. observed that seeking a medical diagnosis of dementia was delayed by about 2 years in half of the cases (4). This may be partly due to the limited knowledge about recognizing the symptoms of dementia and how to access services. Therefore, improving dementia literacy might be an important strategy to increase the possibility of early diagnosis and appropriate management.

Mental health literacy (MHL), first introduced by Jorm et al. in 1997, refers to knowledge and beliefs about mental disorders which aid their recognition, management, or prevention (5). It consists of the following five components: (a) the ability to recognize specific disorders or different types of psychological distress; (b) knowing how to seek mental health information; (c) knowledge of risk factors and causes of self-treatments; (d) knowledge of professional help available; and (e) attitudes that promote recognition and appropriate help seeking (5). In 2009, Low and Anstey adapted MHL to the concept of dementia literacy, which was defined as knowledge and beliefs regarding dementia that aid recognition, management, and prevention (6).

Up to now, studies have reported large variations in dementia literacy even in high-income countries (7–10). In six European countries, the Facing Dementia Survey found that community residents had very limited knowledge of early signs and treatments of Alzheimer’s disease: more than 80% thought most people would not recognize early signs, and 76% thought there were no effective treatments (9). In a study from the UK, the majority of the participants were actively maintaining their health, but still had a poor awareness of the risk factors of dementia (10). In a pilot survey in Australia, 80% of older adults could correctly identify symptoms of dementia, but held widely different views on available treatments and appropriate help-seeking behavior (8).

In contrast, dementia literacy appeared to be much lower in low-resource countries, such as Turkey and Brazil (11, 12). Nearly two-thirds of participants considered some symptoms of dementia as normal aging (11, 12) and ~90% reported that PWD could not stay at home on their own (12).

In China, the general public’s knowledge and understanding of dementia were found to be inconsistent across six studies in five cities: from 14.4% in Shijiazhuang to 78% in Xi’an (13–18). This large difference may partly be explained by the different instruments used to assess dementia literacy. Most studies used self-developed questionnaires (14–18); only Gu and Wang used a validated MHL questionnaire (13, 19). Furthermore, sampling differences also reduce comparability between studies, as some studies selected stratified random sampling (15, 16, 18), some studies chose convenience sampling (17, 19), while other studies did not report their sampling techniques (13, 14). Therefore, it is difficult to reach a consensus on dementia literacy in urban China; and in turn, it is difficult to know how best to include dementia literacy in a national dementia plan.

In this study, we aimed to estimate the dementia literacy in urban China by using a validated assessment instrument and to explore the potential factors associated with the literacy. We anticipated that our study would ultimately provide insight into the population most in need of educational intervention to raise their awareness of dementia diagnosis and care.

## Materials and Methods

### Subject Recruitment

A convenience sample of 3,439 community-dwelling older adults was recruited from 34 cities in 20 provinces (see distribution of the cities and provinces in Tables S1 and S2 in Supplementary Material) between June 20 and August 20, 2014. The trained research assistants in local communities approached the older adults through posters and telephones with the help of the residential committee officers. Those meeting the inclusion criteria were invited for an interview: (1) aged 60 years or above; (2) living in an urban setting; (3) willing to participate in the study; and (4) capable of basic understanding and writing. Those who had other mental disorders, such as schizophrenia or mental retardation, or severe problems of vision, hearing, or speech, were excluded.

The study was approved by the Ethics Review Board of Peking University Institute of Mental Health (Sixth Hospital). Written informed consent was obtained from each participant.

### Instruments

We used selected questions in the MHL questionnaire for older adults to assess the dementia literacy (19). The MHL questionnaire for older adults was developed and validated in China as Zhu and Zhang described (19). Among the 22 questions of the MHL questionnaire, eight questions were related to dementia literacy: four questions on symptoms (Q3–6), one question on the nature of disease (Q1), one question on its prevalence (Q2), one question on the options of appropriate treatment personnel (Q7), and one question on the intention of treatment (Q8) (see the questionnaire in Table S3 in Supplementary Material). Each participant’s general correct response rate (the correct response questions divided by totally answered questions) was computed to indicate the individual’s dementia literacy. Overall dementia literacy of the population was defined as the average correct response rate of each participant.

The clock-drawing test (CDT) was used for screening for cognitive impairment. The CDT is reliable and easy to administer in primary care settings (20). A 4-point scoring method was used. Those with more than two errors had cognitive impairment (21).

The self-reported version of Geriatric Depression Inventory (GDI-SR, 12 items) was used to screen for depressive symptoms as depressive symptoms are a risk factor of dementia (22). The GDI-SR evaluates mood changes in the last 2 weeks, with “yes” (score = 1) or “no” (score = 0) responses. A score of ≥ 3 was categorized as suspected depression (23).

### Statistical Analysis

All statistical analyses were performed with SPSS version 20.0 (SPSS, Inc., Chicago, IL, USA). Pearson’s chi-squared test was used to compare overall dementia literacy among different groups, such as age, gender, schooling educational level, marital status, ethnicity, occupation prior to retirement, per capita annual income, status of cognitive ability, and mood.

Univariate logistic regression analysis was used to test the association of one variable with the dementia literacy without considering other variables or confounders (unconditional association). The dependent variable was defined as the overall level of dementia literacy (0 = low literacy and 1 = high literacy). As reported in the original research of the MHL questionnaire, 0.6 is the recommended cutoff value to divide the participants into “low literacy” and “high literacy” groups (19). The independent variables were defined as follows: gender (0 = male, 1 = female), ethnicity (0 = Han, 1 = other minorities), age (0 = younger than 69 years, 1 = older than 70 years), marital status (0 = not in marriage, 1 = in marriage), occupation (0 = labor work, 1 = non-labor work), mood status (0 = “GDI score ≥ 3”, 1 = “GDI score ≤ 2”), cognitive ability (0 = “CDT score ≤ 2”, 1 = “CDT score ≥ 3”), education (0 = less than 6 years, 1 = more than 6 years), and per capita annual income (RMB) (0 = less than 9,000 CNY, 1 = more than 9,000 CNY).

Due to confounding factors, we implemented multivariate logistic regression analysis, starting with a full model (model with all the variables) and using the stepwise selection approach, where likelihood ratio backward was performed at each step. Standard of enter and remove from the model was 0.10 and 0.15, respectively.

## Results

Among 3,439 participants, 3,007 completed the questionnaire, thus the response rate was 87.4%.

As shown in Table 1, the majority of the respondents aged 60–69 (62.1%), received more than 6 years of education (66.5%), were married (83.1%), were of Han ethnicity (90.9%), and involved in labor work before retirement (81.3%). Most of them had no significant cognitive impairment (77.7%) or suspected depression (63.2%).

**Table 1:** **Group comparisons of overall dementia literacy (*N* = 3,007)**.

Variables	*N* (%)	χ^2^	*p*
**Age**			
60–69 years	1,743 (62.1)	3.261	0.860
≥70 years	1,063 (37.9)		
**Gender**			
Male	1,452 (50.8)	17.592	0.014*
Female	1,407 (49.2)		
**Schooling education**			
≤6 years	956 (33.5)	12.550	0.084
≥7 years	1,895 (66.5)		
**Marital status**			
In marriage	2,369 (83.1)	6.436	0.490
Not in marriage	481 (16.9)		
**Ethnicity**			
Han	2,601 (90.9)	13.919	0.053
Other minorities	259 (9.1)		
**Occupation**			
Labor work	2,030 (81.3)	18.250	0.011*
Non-labor work	466 (18.7)		
**Per capita annual income (RMB)**			
<9,000 CNY	932 (40.9)	77.521	<0.001*
≥9,000 CNY	1,344 (59.1)		
**Cognitive ability**			
CDT score ≤ 2	593 (22.3)	10.468	0.164
CDT score ≥ 3	2,061 (77.7)		
**Mood status**			
GDI score ≥ 3	1,040 (36.8)	15.931	0.026*
GDI score ≤ 2	1,786 (63.2)		

*p < 0.05.

*CDT, clock-drawing test; GDI, Geriatric Depression Inventory*.

The mean overall dementia literacy of all participants was 55.5% (95% CI = 54.7–56.3%, range from 0 to 100%). The overall dementia literacy differed by gender (male < female, χ^2^ = 17.592, *p* = 0.014), occupation prior to retirement (labor work < non-labor work, χ^2^ = 18.250, *p* < 0.011), per capita annual income (χ^2^ = 77.521, *p* < 0.001), and mood status (depression < non-depression, χ^2^ = 15.931, *p* = 0.026). No significant differences were observed between age groups (χ^2^ = 3.261, *p* = 0.860), schooling education (χ^2^ = 12.550, *p* = 0.084), marital status (χ^2^ = 6.436, *p* = 0.490), ethnicity (χ^2^ = 13.919, *p* = 0.053), and cognitive ability (χ^2^ = 10.468, *p* = 0.164) (see Table 1).

As illustrated in Table 2, there was large variation in the single-question correct response rate: lower for the questions on prevalence (Q2: 22.2%) and choosing appropriate professional care personnel (Q7: 22.2%), higher for the questions on intention for treatment (Q8: 67.5%) and symptoms of dementia (Q3–6: 58.7–89.6%). The four questions on symptoms included memory loss as a key symptom (Q3: 89.6%), deficits in activity of daily life (Q4: 59.3%), recent memory and long-term memory deficits (Q5: 61.1%), and an excluding symptom of dementia (Q6: 58.7%).

**Table 2:** **The correct rate of each question of the survey (*N*=3,007)**.

Questions	Correct/total	Correct rate (%)
Q1. The nature of dementia	1,357/2,892	46.9
Q2. Prevalence of dementia	537/2,419	22.2
Q3. Memory loss is a key symptom	2,589/2,888	89.6
Q4. Deficits in activity of daily life	1,708/2,882	59.3
Q5. Recent memory and long-term memory deficits	1,708/2,796	61.1
Q6. Excluding symptom of dementia	1,595/2,719	58.7
Q7. Options of appropriate treatment personnel	605/2,726	22.2
Q8. Intention for treatment	1,943/2,878	67.5

*Correct = correct answered number of people of the item; total = total answered number of people of the item*.

*Correct rate = correctly answered number of people of the item/total answered number of people of the item × 100%*.

As summarized in Table 3, male [OR = 1.298, 95% CI (1.116–1.510)], labor work before retirement [OR = 1.369, 95% CI (1.105–1.696)], low education [OR = 1.208, 95% CI (1.030–1.416)], susceptive cognitive impairment [OR = 1.283, 95% CI (1.040–1.508)], and lower per capita annual income [OR = 1.862, 95% CI (1.563–2.218)] were negatively associated with overall dementia literacy. We did not observe a significant association between the ethnicity, age, marital status, mood status, and overall dementia literacy (both *p* > 0.05). However, in a multivariate stepwise logistic regression analysis, only being male [OR = 1.256, 95% CI (1.022–1.543)], having lower per capita annual income [OR = 1.314, 95% CI (1.064–1.623)], low education [OR = 1.462, 95% CI (1.162–1.839)] and suspected depression [OR = 1.248, 95% CI (1.009–1.543)] were retained as significant negative predictors of dementia literacy.

**Table 3:** **Univariate and multivariate logistic regression analyses of variables associated with dementia literacy**.

Variables	Univariate analysis		Multivariate analysis^#^	
	OR (95% CI)	*p*-Value	OR (95% CI)	*p*-Value
Gender	1.298 (1.116–1.510)	0.001*	1.256 (1.022–1.543)	0.030*
Ethnicity	1.329 (0.917–1.568)	0.064	–	–
Age	1.555 (0.920–1.259)	0.361	–	–
Marital status	1.015 (0.830–1.242)	0.885	1.243 (0.925–1.669)	0.149
Occupation	1.369 (1.105–1.696)	0.004*	–	–
Mood status	1.087 (0.929–1.272)	0.296	1.248 (1.009–1.543)	0.041*
Cognitive ability	1.252 (1.040–1.508)	0.018*	–	–
Education	1.208 (1.030–1.416)	0.020*	1.462 (1.162–1.839)	0.001*
Per capital annual income	1.862 (1.563–2.218)	<0.001*	1.314 (1.064–1.623)	0.011*

*CI, confidence interval; OR, odds ratio*.

*p < 0.05.

*^#^Standard of enter model was 0.10, remove model was 0.15, *N* (case included in multivariate analyses) = 1,745*.

## Discussion

As far as we are aware, our study is the first large-scale survey on dementia literacy among older adults in China’s urban communities. Moreover, we included many important regions, such as Inner Mongolia, Xinjiang, Liaoning, and Guangxi, which were not included in previous studies. Thus, the current study is representative of a relatively broader spectrum of people than previous studies. We observed that, on average, the research participants answered about 55% of the questions correctly. In addition, our study suggested that male, those with lower income, and those with lower education and suspected depression tended to have lower dementia literacy. These important findings not only highlight the need to improve dementia awareness in urban China but also identifies potential groups that health education program should more closely target.

In our study, the overall dementia literacy is 55.5%, i.e., the participants only knew half of the knowledge about dementia tested. This level is almost similar to that in Shanghai (54%), 10 years ago which used the same instrument tool with ours (13). This may be explained as Shanghai is one of the first aging cities in China (24), and there has been lots of education since late 1990s.

As we expected, the majority of older adults in our study knew symptoms of dementia and expressed intention for treatment. However, the majority (78%) of the respondents underestimated the prevalence of dementia. This could be partly due to their misunderstanding of the nature of dementia. More than half of the respondents believed that “dementia is part of normal aging.” This belief was shared by Latino and African-American older adults as well as dementia caregivers in China (15, 25–30). In addition, biomarkers for early diagnosis of dementia are yet to be validated (31). In milder cases, functional impairment may be too subtle to be detected solely through observations by lay persons (32). This may further hinder the understanding of the facts of dementia.

In our study, the rate of intention for treatment was high (nearly 70%), while only one-fifth did not choose traditional practitioners for professional help. With regard to aged care and chronic disease treatment, older Chinese are skeptical of the safety of Western medicine and favor traditional medicine in preference to specialist care (33). We found that even in well-established memory clinics, only a small portion of family caregivers of PWDs received training on dementia care (34). The families sought medical help about 2 years after they noticed symptoms of cognitive impairment (4). These findings suggest that literacy about choosing optimal professional care personnel remains poor. More efforts should be invested to improve accessibility to dementia care services.

The present study demonstrated that women had a better knowledge about dementia compared to men. This observation is consistent with the previous study by Arai et al (35). In Chinese culture, women tend to be more involved in family caregiving than men, and thus pay more attention to health issues including dementia. Women may also play an active role in community management and volunteer services that may create more opportunities to acquire and share knowledge of symptoms of dementia.

Consists with studies by Lee et al. and von dem Knesebeck et al., education emerged as the most important factor influencing literacy. Lower level of education negatively correlates with dementia literacy. The results of the present study provide empirical evidence that education can be helpful in increasing dementia literacy. Highly educated individuals tend to know more about the symptoms of mental disease than the less educated individuals do (36, 37).

In contrast, the relationship between age and knowledge about dementia remains inconsistent. Some found that older age was associated with less knowledge (35, 36, 38, 39). Others found younger participants were less likely to be knowledgeable (40). This may be explained by methodology differences. Our study only included older adults, rather than public of all ages.

We also found that annual income was negatively associated with overall literacy. Low socioeconomic status may restrict access to health education resources, especially in low-resources areas. Consistent with Tan et al., we suggest that special support programs for low-income people are imperative to improve their knowledge about dementia (41).

Few studies have looked into the effect of mental health status on dementia literacy. Our study found that participants with depressive symptoms have relatively low literacy compared with those without depressive symptoms. After adjusting for potential covariates, depression remains an independent factor of overall literacy. When suffering from depression, older adults tend to reduce participation in community activities, become less interested in social engagement, and thus, decrease the opportunities to gain knowledge (42, 43). Second, older Chinese with depression pay more attention to somatic discomforts. Due to poor concentration and motivation, the retrieval of acquired knowledge may be compromised (44). This finding emphasizes the important role of mental well-being in health education and knowledge dissemination.

Our study had certain limitations that may influence our results. Due to bias in the convenience sample, older adults who consented to the interview may be more active in community participation. As homebound and socially isolated older adults were not recruited, the overall dementia literacy might be overestimated. Hence, the results should be interpreted with caution. However, the age and gender composition in our survey were similar to that of the national population according to the sixth National Population Census. Therefore, this survey might have a relatively high representation (see Tables S4 and S5 in Supplementary Material) (45). Second, we did not inquire about whether participants had joined the health educations available in local settings. Thus, it is difficult to answer whether increasing educational resources would promote dementia literacy in the community. Third, one possible explanation of the low dementia literacy found in the cognitive impairment group is that the cognitive decline of these patients made it difficult for them to understand the relevant questions. To minimize the potential bias of cognitive impairment, we used face-to-face interviews.

Our study was designed to describe overall dementia literacy among community-living older adults living in urban areas, using a validated instrument. It shows that although older Chinese have some knowledge about symptoms of dementia, they have poor literacy about the impact of the disease and choosing optimal professional care personnel. We also found that the following risk factors were associated with lower dementia literacy: men, lower income, and those with suspected depression. These findings suggest that overall dementia literacy remains low and educational interventions should be prioritized in the community. Therefore, it will be of great significance to advocate for improving dementia literacy in the future national dementia plan, such as awareness raining campaigns.

## Conclusion

Our study is the first large-scale survey on dementia literacy among community-dwelling older adults in urban China. It reveals the knowledge gaps about dementia: older Chinese know more about symptoms than the impact and care resources. It also identifies the factors influencing dementia literacy: gender, income, education, and mood status. Further educational programs should not only focus on symptoms but also provide dementia care resources, including memory clinics. In addition, specific population groups such as men, lower income, lower education, and family members of people with suspected depression should be targeted for health education activities in the community. This also poses interesting questions of how well-trained doctors are to relate to these groups and how those with a concern about their memories will access an assessment for dementia in a community health center or elsewhere.

## Ethics Statement

This study was carried out in accordance with the recommendations of Ethical Review Guideline, the Ethics Review Board of Peking University Institute of Mental Health (Sixth Hospital) with written informed consent from all subjects. All subjects gave written informed consent in accordance with the Declaration of Helsinki. The protocol was approved by the Ethics Review Board of Peking University Institute of Mental Health (Sixth Hospital).

## Author Contributions

HW and XY formulated the research questions. HZ, MZ, XL, HW, and XY designed the study and supervised the data collection. HZ, SZ, MZ, XW, JW, XL, and HW collected the data. HZ, SL, MZ, XL, NL, and HW carried out the statistical analysis. HZ, SL, XL, NL, and HW wrote the paper. XY provided critical review and comments on the manuscript.

## Conflict of Interest Statement

The authors declare that the research was conducted in the absence of any commercial or financial relationships that could be construed as a potential conflict of interest.
