# Supplementary material for: Dementia Literacy among Community-Dwelling Older Adults in Urban China: A Cross-sectional Study
Source: Front Public Health. 2017 Jun 7;5:124. doi: 10.3389/fpubh.2017.00124 (PMC5461251; doi:10.3389/fpubh.2017.00124)
Supplement: Supplementary file 1 [file Table_1.PDF]

Table S1. Distribution of the Provinces in the survey

Table S1. Distribution of the Provinces in the survey

Name of Provinces	Sample size	Percentage
Beijing	413	0.14
Fujian	60	0.02
Gansu	179	0.06
Guangdong	136	0.05
Guangxi	118	0.04
Guizhou	63	0.02
Hebei	283	0.09
Henan	146	0.05
Hubei	65	0.02
Hunan	26	0.01
Jilin	60	0.02
Jiangsu	120	0.04
Jiangxi	102	0.03
Liaoning	319	0.11
Inner Mongolia	308	0.10
Shandong	246	0.08
Shanxi	182	0.06
Tianjin	63	0.02
Xinjiang	60	0.02
Zhejiang	58	0.02
Sum	3007	1.00
