# Supplementary material for: Dementia Literacy among Community-Dwelling Older Adults in Urban China: A Cross-sectional Study
Source: Front Public Health. 2017 Jun 7;5:124. doi: 10.3389/fpubh.2017.00124 (PMC5461251; doi:10.3389/fpubh.2017.00124)
Supplement: Supplementary file 2 [file Table_2.PDF]

Supplemental Table S2. Distribution of the cities in the survey

Table S2. Distribution of the cities in the survey

City	Provinces	Sample size	Percentage
Beijing	Beijing	413	0.14
Xiamen	Fujian	60	0.02
Lanzhou	Gansu	120	0.04
Linxia	Gansu	59	0.02
Dongguan	Guangdong	52	0.02
Shenzhen	Guangdong	84	0.03
Liuzhou	Guangxi	62	0.02
Nanning	Guangxi	56	0.02
Bijie	Guizhou	63	0.02
Baoding	Hebei	60	0.02
Handan	Hebei	77	0.03
Zhangjiakou	Hebei	146	0.05
Puyang	Henan	99	0.03
Zhengzhou	Henan	47	0.02
Enshi	Hubei	65	0.02
Yueyang	Hunan	26	0.01
Changchun	Jilin	60	0.02
Nanjing	Jiangsu	120	0.04
Nanchang	Jiangxi	42	0.01
Shangrao	Jiangxi	60	0.02
Anshan	Liaoning	60	0.02
Dalian	Liaoning	128	0.04
Tieling	Liaoning	60	0.02
Yingkou	Liaoning	71	0.02
Baotou	Inner Mongolia	43	0.01
Chifeng	Inner Mongolia	265	0.09
Liaocheng	Shandong	120	0.04
Yantai	Shandong	66	0.02
Zibo	Shandong	60	0.02
Jincheng	Shanxi	60	0.02
Yuncheng	Shanxi	122	0.04
Tianjin	Tianjin	63	0.02
Urumqi	Xinjiang	60	0.02
Ningbo	Zhejiang	58	0.02
Sum		3007	1
