# Supplementary material for: Dementia Literacy among Community-Dwelling Older Adults in Urban China: A Cross-sectional Study
Source: Front Public Health. 2017 Jun 7;5:124. doi: 10.3389/fpubh.2017.00124 (PMC5461251; doi:10.3389/fpubh.2017.00124)
Supplement: Supplementary file 3 [file Table_3.PDF]

Supplemental Table S3. The questionnaire o f dementia literacy

Table S3. The questionnaire of dementia literacy

1. In the following opinions related to dementia, which one you do not agree?	1 = dementia is a disease ,<br>2 = dementia is not a disease, it is a normal aging process ,<br>3 = dementia is common among elderly ,<br>4 = dementia does not mean a disease, but rather refers to a variety of disease
2. What is the prevalence rate of dementia?	1=5/100000,<br>2=5/10000,<br>3=5/1000,<br>4= 5/100
3. In the following options, which one is likely to be a symptom of dementia	1 = Headache<br>2=faint<br>3= dizziness<br>4 = bad memory
4. Which is the most common difficulty would people with dementia meet?	1 = learning difficulty<br>2 = difficulty in work<br>3 = difficulty in self-care<br>4=all of above
5.Which is the most common symptom of dementia:	1 = it is easier to forget the recent events than the past events,<br>2= it is easier to forget the past events than the recent events,<br>3= it is easy to forget both past events and recent events,<br>4= none of above
6.Which of the following is not a symptom of dementia:	1 = patients can get lost easily,<br>2=Patients cannot remember someone's name,<br>3= Patients often forget to return the things that they borrowed back,<br>4= patients do not forget their own things
7.If you or your family members get dementia, which doctor you would not seek for help	1 = internists,<br>2 = Neurologists,

	3 = Traditional Chinese Medicine Doctors,<br><br>4 = psychiatrist
8. In the following opinions related to dementia, which one do you agree?	1 = Dementia is not a disease, treatment is not needed,<br><br>2 = although dementia is a disease, treatment is not necessary,<br><br>3= Dementia cannot be cured, treatment is not needed,<br><br>4= Dementia is a disease, treatment is needed
