# Supplementary material for: Dementia Literacy among Community-Dwelling Older Adults in Urban China: A Cross-sectional Study
Source: Front Public Health. 2017 Jun 7;5:124. doi: 10.3389/fpubh.2017.00124 (PMC5461251; doi:10.3389/fpubh.2017.00124)
Supplement: Supplementary file 4 [file Table_4.PDF]

Table S4. The comparison of the proportion of the sample and the overall population

Table S4. the comparison of the proportion of the sample and the overall population

Age	Overall number	Percentage	Sample number	Percentage
60-64	58667282	0.330	1077	0.363
65-69	41113282	0.232	712	0.240
70-74	32972397	0.186	534	0.180
75-79	23852133	0.134	404	0.136
>=80	20989346	0.118	241	0.081
SUM	177594440	1	2968	1

Reference data, http://www.stats.gov.cn/tjsj/pcsj/rkpc/6rp/
