# Supplementary material for: Dementia Literacy among Community-Dwelling Older Adults in Urban China: A Cross-sectional Study
Source: Front Public Health. 2017 Jun 7;5:124. doi: 10.3389/fpubh.2017.00124 (PMC5461251; doi:10.3389/fpubh.2017.00124)
Supplement: Supplementary file 5 [file Table_5.PDF]

Table S5. The comparison of the percentage   gender between sample and the overall of population

Table S5. the comparison of the percentage of gender between sample and the overall population

Gender	Male	Female	$\chi2$	*p*
Sample	1529	1474	4.000	0.261
Overall	87031109	90527397		

Reference data, http://www.stats.gov.cn/tjsj/pcsj/rkpc/6rp/
